# Prevalence of Over-the-counter use of Antibiotics among Patients visiting Outpatient Department of a Primary Health Centre: An Observational Study

**DOI:** 10.31729/jnma.v64i293.9280

**Published:** 2026-01-31

**Authors:** Shova Sapkota, Kiran Shrestha, Parikshit Prasai, Mausam Thapa Magar, Ojaswee Karki

**Affiliations:** 1 Melamchi Primary Health Centre, Melamchi, Sindhupalchok, Nepal; 2 Parbat District Hospital, Badagaoon Sadak, Kushma, Nepal; 3 Kathmandu Medical College, Sinamangal, Kathmandu, Nepal

**Keywords:** *antibiotics*, *drug resistance*, *microbial*, *self-medication*

## Abstract

**Introduction:**

Antimicrobials are the most commonly used medicine. Over-the-counter use of antibiotics is more prevalent in developing countries, contributing to inappropriate use and increasing antimicrobial resistance. Studies regarding over the counter use of antibiotics are lacking in Nepal. The objective of this study was to determine prevalence of over the counter use of antibiotics among patients visiting primary health centre.

**Methods:**

A descriptive cross-sectional study was conducted among 377 participants from October 2024 to February 2025 at Outpatient department of Melamchi Primary Health Centre after obtaining ethical approval from the Ethical Review Board of Nepal Health Research Centre (Reference no: 820). Written informed consent was obtained and prevalence of over-the-counter use of antibiotics was examined using a structured proforma. Data was entered into Microsoft Excel, and descriptive statistics were calculated.

**Results:**

Among 377 participants, 86 (22.81%) were using over-the-counter antibiotics, of which 45 (52.32%) were male and 41 (47.67%) were female. 40 (46.51%) of those participants using over-the-counter antibiotics were older than 15 years, whereas 3 (3.48%) were less than 1 year of age. The most common presenting symptoms in these patients were related to the respiratory system 39 (45.34%). The most commonly used antibiotic was Amoxicillin plus clavulanic acid; 32 (37.20%).

**Conclusions:**

The study reported a lower prevalence of over-the-counter use of antibiotics among patients presenting to primary health centers compared to the ‘WHO South-East Asia Region’.

## INTRODUCTION

Antimicrobials are the most commonly used drugs worldwide.^1^ Over-the-counter use of antibiotics may lead to masking of symptoms, drug interactions, and antimicrobial resistance.^2^ Prevalence of over-the-counter use of antibiotics is higher in developing countries due to the easy availability of antibiotics, poor supervision, and control of antibiotic selling.^3^ The overall prevalence of over-the-counter use with antibiotics in the ‘WHO South-East Asia Region’ is 42.64% with a higher prevalence in Nepal and India.^2^ Substantial use of antibiotics from informal sectors in low and middle-income countries accounts for low antibiotics use in reports from health databases.^4^

Though few studies have been conducted among nurses and medical students, community-based studies on over the counter use of antibiotics are still lacking in Nepal.^5^ This study aimed to determine prevalence of over-the-counter use of antibiotics among patients visiting primary health centers.

## METHODS

A descriptive cross-sectional study was conducted at the outpatient department of Melamchi Primary Health Centre, Melamchi, Nepal from October 2024 to February 2025. Ethical approval was obtained from the Ethical Review Board of the Nepal Health Research Council ‘(Reference No: 820)’. Informed written consent was obtained from participants above 18 years of age. The written consent was obtained from parents of participants less than 18 years. Additional written assent and verbal consent was taken from participants of 12 to 18 years and 7 to 12 years respectively.

Patients presenting with symptoms of infection such as fever, cough, sore throat, loose stool, burning micturition etc were included in the study. Patients presenting for follow-up of infectious diseases within seven days, and patients taking prophylactic antibiotics for Rheumatic heart disease or infective endocarditis were excluded from the study.

A convenience sampling technique was used. Sample size was calculated using a formula:

n = required sample size
Z = 1.96 at 95% Confidence Interval (CI)
p = prevalence, 42.64% - prevalence of self-medication with antibiotics in ‘WHO South-East Asia Region’^2^
e = margin of error, 5%

$$\begin{array}{ll} \text{n} & ={\text{Z}}^{2}\times \frac{\text{p}\times (1-p)}{{\text{e}}^{\text{2}}}\\ & =1.96^{2}\times \frac{0.4264\times 0.5754}{0.05^{2}}\\ & \begin{array}{l} =377.01\\ =377 \end{array} \end{array}$$

Data collection was done using a “pre-designed proforma”. Obtained data were entered in Microsoft Excel and analyzed using Statistical Package for the Social Sciences (SPSS) software version 26.

## RESULTS

Among 377 participants, 186 (49.33%) were male and 191 (50.66%) were female. The prevalence of over-the-counter use of antibiotics among participants was found to be 86 (22.81%). Among those who used antibiotics over the counter, 45 (52.32%) and 41 (47.67%) were male and female respectively.

**Table 1:** Age-wise distribution of participants using over-the-counter antibiotics (n=86).

Age group (Years)	n(%)
Less than 1	3(3.48)
1-5	19(22.09)
6-15	24(27.90)
More than 15	40(46.51)

**Table 2:** Antibiotics used by participants using over-the-counter antibiotics (n=86).

Antibiotics used	n(%)
Amoxicillin	14(16.27)
Amoxicillin plus clavulanic acid	32(37.20)
Azithromycin	6(6.97)
Cefixime	16(18.60)
Cefpodoxime Proxetil	5(5.81)
Cloxacillin	3(3.48)
Flucloxacillin	1(116)
Metronidazole	2(2.32)
Combination of antibiotics	7(8.13)

**Figure 1:**
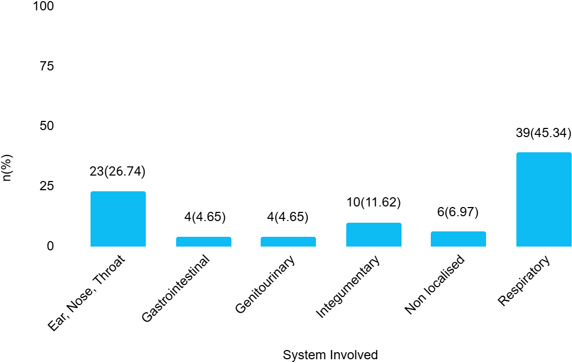
Systems involved in participants using over the counter antibiotics based on symptoms (n=86).

Among participants using over the counter antibiotics, 39 (45.34%) presented with symptoms of respiratory system involvement such as cough, chest pain, and fever (Figure 1).

Amoxicillin plus clavulanic acid was the most commonly used antibiotic while 7 (8.13%) participants used a combination of antibiotics, (Table 2). Among seven patients each of them used combination of amoxicillin plus clavulanic acid with azithromycin, cloxacillin, flucloxacillin, metronidazole, and albendazole, amoxicillin and azithromycin, and two participants used a combination of Azithromycin and Cefpodoxime. Three of these combinations of Amoxicillin plus Cloxacillin, Azithromycin plus Cefpodoxime, and Azithromycin plus Cefixime have been listed as Not Recommended combination by WHO AWaRe classification 2023.^6^

**Figure 2:**
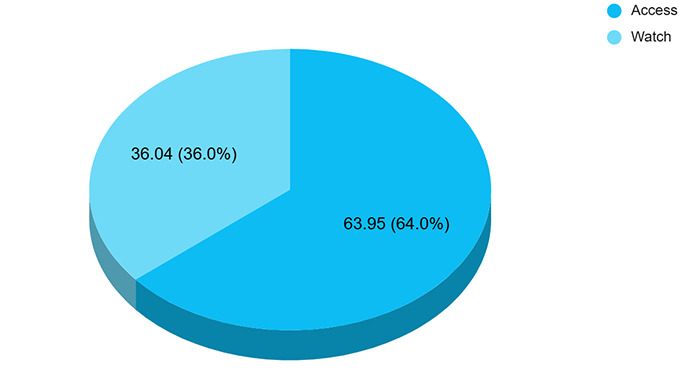
Class of antibiotics used by participants as per WHO AWaRe classification 2023 (n=86).

Among the antibiotics used over the counter 55 (63.95%) and 31 (36.04%) were in the ‘Access’ and ‘Watch’ class of WHO AWaRe classification 2023 (Figure 2).^6^

## DISCUSSION

This study revealed that 22.8% (86 out of 377) of participants reported using antibiotics obtained over-the-counter without a prescription. This prevalence was lower than that reported in two systematic reviews conducted in low-and middle-income countries (LMICs), in which over-the-counter use of antibiotics ranged from 38.8% to 62%, depending on various factors such as healthcare accessibility and geographic location.^7,8^ In a community-based study in Islamabad, Pakistan, the prevalence was 32.5%. Similarly, studies performed in other developing countries, such as Kenya, Nigeria, Yemen, and Saudi Arabia, showed 60.0%, 82.2%, 87.1%, and 40.8% prevalence of over-the-counter use of antibiotics respectively which is much higher compared to our study.^9^ In a cross-sectional study conducted in 25 pharmacies to check the status of antibiotic dispensing in Kathmandu valley, the prevalence of people using antibiotics without prescription was found to be 56%.^10^ And in a study done in the emergency department of a tertiary care center in Nepal, 70.38% of people used over-the-counter antibiotics before visiting the tertiary care center, which is also higher than in our study.^11^

In our study, over-the-counter antibiotic use was nearly equal in distribution among males and females with 52.3% and 47.7% respectively, which is consistent with previous findings that suggested no significant gender bias in over-the-counter antibiotic use practices. In a study conducted in Iran, there were 40.48% male users and 59.52% female users.^12^ In a study conducted in various European countries, approximately 42%-69% (Mean 55.5%) of over-the-counter antibiotic users were female.^13^ In a study done in Bangladesh over 50% of the patients were between the ages of 21-30 years, with 83.57% of them being males and 16.43% females.^3^

In our study, about half the population (46.5%) of over-the-counter antibiotic users were older than 15 years, however, the other half (53.5%) of over-the-counter antibiotic users were children aged 1-15 years, which is concerning as this reflects parental administration and indicates a need for targeted public education about antimicrobial resistance and other side effects of inappropriate use of antibiotics in children.^14^ In a study conducted in southern China, most (47.8%) users were above 18 years, which is similar to our study.^15^ In a study conducted in a primary care center in Ankara, the highest prevalence was seen in the 40-49 age group (23%), lowest in the 60-69 age group (11.8%).^16^ A study shows that there is a higher prevalence of over-the-counter antibiotic use among children in the Middle East (34%), Africa (22%), Asia (20%) and South America (17%), while the lowest prevalence was found in Europe (8%).^17^

Our study revealed that respiratory symptoms (45.3%), such as cough, chest pain, and fever, were the most common reasons for over-the-counter antibiotic use, followed by ENT illnesses (26.7%). In similar studies, the most common reasons for over-the-counter antibiotic use were observed to be sore throat (59.6%), fever (46.2%), and cough (40.0%), which is similar to our study.^16^ This pattern depicts the misconception of antibiotics being effective against any illnesses including viral infections which are self-limiting and not affected by antibiotics treatment.^18^ This rampant and irrational use of over-the-counter antibiotic is contributing to the global antimicrobial resistance threat.^1^

In our study, amoxicillin-clavulanic acid was the most commonly chosen antibiotic (37.20%), followed by cefixime (18.60%) and amoxicillin (16.27%). This trend is also similar to previous studies performed in other countries, which showed overuse of broad-spectrum antibiotics, especially from the “Watch” group in the WHO AWaRe classification.^19^ In a survey of antibiotic dispensing patterns done in Nepal, 38.4% of the people visiting pharmacies received at least one antibiotic which was above the WHO recommended value (20.0%-26.8%). The most commonly dispensed antibiotics were cefixime (16.9%), amoxicillin (12.2%), cefpodoxime (10.3%), ampicillin + cloxacillin (8.7%) and ciprofloxacin (8.7%).^20^ What is most concerning is that 8.1% of the participants in our study reported the use of combinations of antibiotics, some of these combinations are classified as ‘Not Recommended’ by the WHO AWaRe classification 2023.^6^ The use of such combinations lacks synergistic benefits, increases likelihood of side effects and increases risk of antimicrobial resistance. Antibiotic resistance remains as threat to public health by increasing the cost of treatment and mortality which affects the economy as a whole.^19^

However, regulatory efforts and antibiotic dispensing laws are often weak in many countries, resulting in easy availability and accessibility of these antibiotics.^1^ Hence, strict law enforcement and proper pharmacy regulations are required to prevent people from easily accessing these antibiotics, along with public awareness campaigns and pharmacist training.^21^

## CONCLUSION

Our study demonstrated a lower prevalence of over-the-counter use of antibiotics among patients presenting to Primary Health Centre compared to the ‘WHO South-East Asia Region’ and a higher prevalence of over-the-counter use of the 'Watch' class of WHO AWaRe criteria. Further studies in a community and a tertiary care setting are warranted. Implementation of strict antimicrobial stewardship policies is recommended.

## Data Availability

The data are available from the corresponding author upon reasonable request.
